# Neonatal Extracorporeal Membrane Oxygenation Due to Respiratory Failure: A Single Center Experience Over 28 Years

**DOI:** 10.3389/fped.2018.00263

**Published:** 2018-09-25

**Authors:** Friedrich Reiterer, Elisabeth Resch, Michaela Haim, Ute Maurer-Fellbaum, Michael Riccabona, Gerfried Zobel, Berndt Urlesberger, Bernhard Resch

**Affiliations:** ^1^Division of Neonatology, Department of Pediatrics and Adolescent Medicine, Medical University of Graz, Graz, Austria; ^2^Research Unit for Neonatal Infectious Diseases and Epidemiology, Medical University of Graz, Graz, Austria; ^3^Outpatient Clinic of Neurodevelopmental Follow-Up, Division of Neonatology, Department of Pediatrics and Adolescent Medicine, Medical University of Graz, Graz, Austria; ^4^Division of Pediatric Radiology, Department of Radiology, Medical University of Graz, Graz, Austria; ^5^Pediatric Intensive Care Unit, Department of Pediatrics and Adolescent Medicine, Medical University of Graz, Graz, Austria

**Keywords:** ECMO complications, neonatal ECMO, neurodevelopmental outcome, respiratory failure, survival rate

## Abstract

**Background:** ECMO therapy is worldwide declining in the neonatal population; hence, its therapeutic value is sometimes questioned.

**Objectives:** To report our experience with neonatal ECMO due to respiratory failure over a 28 year time period.

**Methods:** Retrospective single center observational study including all neonates admitted to ECMO due to respiratory failure between 1989 and 2016 at Graz, Austria. Data were collected regarding survival rate, duration of ECMO, complications, length of hospital stay, changes over time, and follow-up.

**Results:** Sixty-seven neonates were admitted and 43 (64%) needed ECMO—median birth weight 3390 grams (range 1810–4150) and gestational age 39 weeks (32–43). Survival rate was 65% (28/43); with higher rates in meconium aspiration syndrome (MAS) 89% vs. congenital diaphragmatic hernia (CDH) 46% and septic shock 44% (*p* = 0.005 and *p* = 0.006, respectively). ECMO duration was median 5 days (1–30) and veno-arterial ECMO (52%) dominated. Need for ECMO therapy decreased over time (*p* < 0.001). Complications occurred in 31 (72%) neonates. Five neonates had cerebral hemorrhages (11.4%) and four had cerebral infarction (9.1%). Of 26 survivors 17 (65%) showed normal neurodevelopmental outcome at median follow-up of 73 months. Motor deficits were present in one case, cognitive deficits in 9 (35%). Median length of hospital stay was 78 days in those with deficits and 29 in those with normal neurodevelopmental outcome (*p* < 0.001).

**Conclusions:** Survival rate did not change over the study time but indications for ECMO did. Cognitive impairment was the major long-term deficit following neonatal ECMO being associated with longer hospital stay.

## Introduction

Extracorporeal Membrane Oxygenation (ECMO) was introduced in the early 1970's as a rescue therapy of acute cardiorespiratory failure unresponsive to conventional treatment strategies. Thereafter, ECMO soon became a successful life-saving intervention in term and near-term infants with diagnoses including severe meconium aspiration syndrome (MAS), respiratory distress syndrome (RDS), congenital diaphragmatic hernia (CDH), sepsis, post-operative cardiac failure, and persistent pulmonary hypertension of the neonate (PPHN) (1). In 1989, the International Extracorporeal Life Support Organization (ELSO) was founded connecting centers which were actively using extracorporeal life support (ECLS/ECMO). Its primary intention was to maintain an international registry of ECLS cases providing standardized data collection forms with the goal to improve outcomes and reduce adverse events; and on the other hand to publish guidelines for ECLS. In 2016, the ELSO reported a total of 36.964 neonatal cases (2). The registry mainly presented cases of respiratory failure formatting the single largest cumulative cohort of patients (37%). In those patients, the survival rate to hospital discharge was 74% (2). In the last two decades, the use of neonatal ECMO for respiratory (but not cardiac) failure decreased worldwide due to improvements in neonatal intensive care medicine including advances in mechanical ventilation (MV), availability of surfactant therapy for RDS and inhaled nitric oxide (iNO) for PPHN.

Aim of this study was to report our experience with neonatal ECMO due to respiratory failure over a 28 year time period including long-term neurodevelopmental outcome data.

## Patients and methods

All neonates admitted to ECMO therapy due to respiratory failure between 1989 and 2016 at the neonatal intensive care unit (NICU) of the Division of Neonatology of the Pediatric Department of the Medical University of Graz, Austria, were included for retrospective analysis. The study was approved by the local ethic committee (27–432 ex 14/15). Data collection started in the beginning of 2017. Postoperative cardiac patients were not included. The medical charts and the data from our Outpatient Clinic of Neurodevelopmental Follow-up were reviewed. Perinatal data included birth weight (BW) in grams, gestational age (GA) in weeks, Apgar scores at 1, 5, and 10 min, umbilical artery ph, and history of intern or extern referral. Respiratory diagnoses were collected, and data on ECMO including veno-arterial (VA) or veno-venous (VV) ECMO, start with ECMO therapy in hours (from admission to ECMO initiation), duration in days, complications, number of survivors without ECMO, number lost to follow-up, and deaths (before, during, or following ECMO). Every neonate received pre- (as far as congenital heart disease and cranial bleedings remained contradictions for ECMO treatment) and post-ECMO cranial ultrasound (CUS); and in case of any pathologic findings cranial MRI was performed (over the last decade).

*ECMO entry criteria*, which did not change over time, were as follows: GA of ≥ 34 weeks or BW ≥ 2000 g, respiratory failure despite maximum conventional therapy including high frequency oscillatory ventilation (HFO), iNO and surfactant application, no severe coagulopathy, no larger bleeding, duration of mechanical ventilation (MV) < 14 days, reversible pulmonary disease, no hemodynamically significant congenital heart disease, and no major congenital malformation/abnormality (excluded CDH). Respiratory criteria included high oxygenation indices (OI) with values of > 40 persisting for at least 4–6 h (OI = mean airway pressure [MAP] × FiO_2_/PaO_2_) or high values of Arterio-alveolar Difference of oxygen (AaDO2 > 600). In presence of a severe and persistent air leak initiation of ECMO was accelerated.

## ECMO-system and cannulation

Our initial neonatal ECMO- system consisted of a servo-controlled occlusive roller pump (Stockert-Shiley, Munich, Germany), a membrane oxygenator with silicone membrane lungs of either 0.4 or 0.8 m^2^ membrane surface area (Avecor Cardiovascular Inc, Plymouth, MN), a special 0.25 inch silicon rubber tubing with a small reservoir in the venous drainage part, a heat exchanger, an electronically controlled gas flowmeter, a pressure monitoring unit and sensors for air bubbles and temperature. Since 2011, we used a new ECMO-system with a centrifugal non-occlusive pump (Bio-Medicus™, Medronic Austria). Cannulation was exclusively performed by an experienced cardiac surgeon under general anesthesia. For a veno-arterial bypass (VA-ECMO), the right internal jugular vein and common carotid artery were cannulated by appropriate seized catheters. To avoid ligation of the common carotid artery we applied an arterial cannulation technique using a Gore-Tex graft. This part of the graft also provided a patch at the arteriotomy after decannulation (3). Veno-venous bypass (VV-ECMO) was performed in most cases via the internal jugular vein and right femoral vein. VA-ECMO was preferentially used in our first ECMO-patients and later reserved for patients with severe cardiorespiratory failure. Catheter positioning was sonographically guided to avoid or minimize catheter-associated complications like drainage problems due to catheter malposition (4). Anticoagulation during ECMO was achieved by continuous heparin infusion up to 30 IE/kg/h and monitored by measuring regularly (twice a day) the activated clotting time (ACT), targeted between 150 and 200 s depending on the individual bleeding risk of a patient (5). Other coagulation parameters were also monitored routinely as consumptive coagulopathy frequently occurred during ECMO, especially within the first 24 h. For a detailed description of the physiology and clinical use of neonatal ECMO, we refer to the literature (6).

*Complications during ECMO* therapy were classified as: mechanical, hemorrhagic, and others.

For trends (e.g., survival rate over time) the study time period was divided into 3 time spans: T1 from 1989 to 1997, T2 from 1998 to 2007, and T3 from 2008 to 2016.

For *neurodevelopmental outcome* infants were assessed at the age of 4, 8, 12, 18, and 24 months, then once a year (up to school age) and thereafter individually (without follow-up protocol). Assessment of outcome was made using the developmental tests as described by Griffith for the first 2 years followed by Kaufman after these years. Within the last decade using Bayley Scales of Infant Development, Wechsler Preschool and Primary Scale of Intelligence, and neurological examinations as described by Amiel-Tison and Touwen were used (7–12). Classification of mental outcome included normal, developmental delay, and mental retardation. Classification of neurological outcome included normal, minimal cerebral dysfunction (including dystonia, hypotonia, and asymmetry), cerebral palsy and ataxia. Classification of visual disorders included strabismus and visual impairment, classification of hearing disorders hearing impairment. Behavioral disabilities were collected in a descriptive way.

*Statistical analyses* were performed using the *t*-test and Wilcoxon test for numerical data and the x^2^ test using Yates correction and Fisher's exact test as appropriate for categorical data. For all statistical tests, a level of significance of 0.05 was used. Descriptive analyses were done using Microsoft Excel 2010 (Microsoft Corporation, 2010, Redmond, USA), all other tests by using SPSS 22 (IBM SPSS Inc, Chicago, Illinois, USA, 2014).

## Results

**Figure 1:**
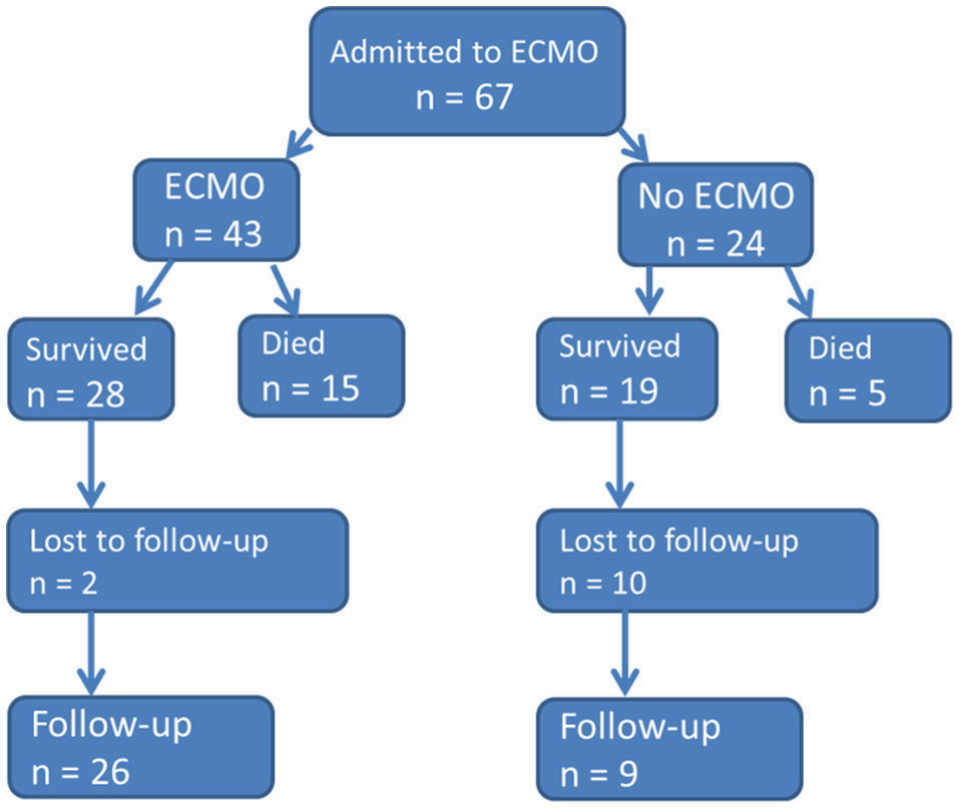
Flow chart of neonatal ECMO patient recruitment between 1989 and 2016.

During the study period a total of 67 neonates were admitted due to respiratory failure to our neonatal ECMO center at the NICU (see flowchart in Figure 1).

### Perinatal history

**Figure 2:**
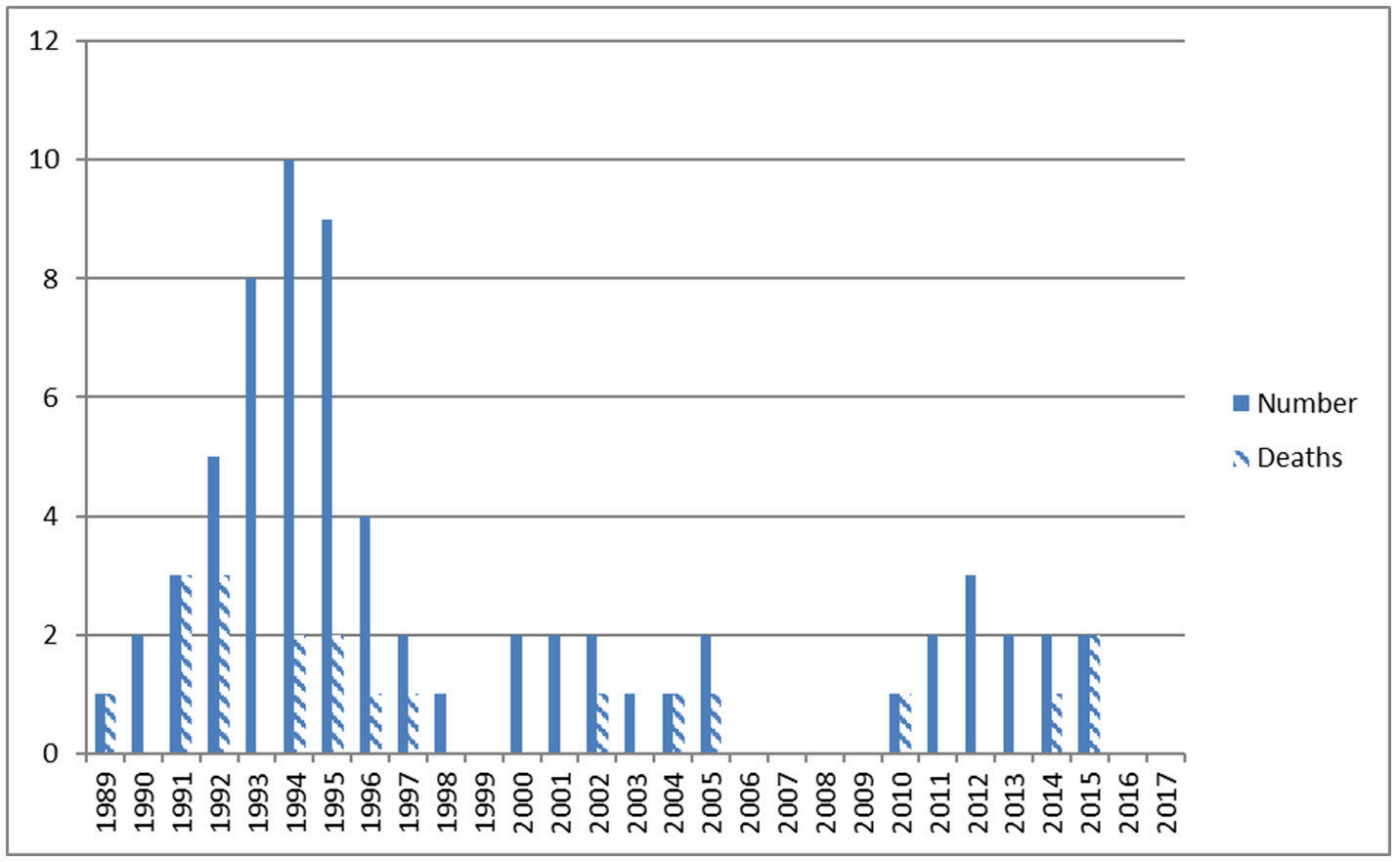
ECMO admissions and number of deaths between 1989 and 2016.

Perinatal data are given in Table 1. Admissions per year and deaths before, during, and following ECMO therapy are shown in Figure 2. Diagnoses included MAS (*n* = 25), CDH (*n* = 17), early-onset sepsis (EOS, *n* = 15), PPHN (*n* = 6), lung hypoplasia (*n* = 3), and neonatal varicella with respiratory failure (*n* = 1). In 40 cases (60%) pregnancy including fetal sonography was unremarkable. Perinatal history included no events in 14 (21%), meconium stained amniotic fluid in 29 (43%), and fetal distress in 34 (51%). Preterm rupture of the membranes and chorioamnionitis occurred in 5 (7.5%) cases. Postpartum 41 neonates (61%) were immediately intubated. MV started at a mean age of 13 h (1–336), HFO was initiated in 42 (63%) and iNO in 46 (69%) neonates. Hemodynamic support was given to 46 (69%) neonates. Maximum mean (±SD) OI was 36 ± 15, AaDO2 was 599 ± 47, FiO2 was 0.95 ± 0.15 and pCO2 83 ± 36 mmHg. Seventeen cases were from our Institution (25%), 26 were referred from level 3 (39%), 8 from level 2 (12%), and 16 from level 1 hospitals (24%).

**Table 1:** Perinatal data of 67 neonates admitted for ECMO therapy between 1989 and 2016.

**Perinatal parameter**	**Number**
Birth weight (in grams)	3216 ± 546
Gestational age (in weeks)	39 ± 3
Apgar score at 1 min	5, 6 ± 2, 8
Apgar score at 5 min	7, 2 ± 2, 1
Apgar score at 10 min	7, 9 ± 1, 9
Umbilical artery pH	7, 22 ± 0, 11
Intern referral	18 (27)
Extern referral	49 (73)

*Data are given as n (%) or mean ± SD*.

### Survival rate

Five of 67 neonates (7.5%) died before cannulation to ECMO, and 19 (28%) did not need ECMO. Within the remaining group of 43 neonates 12 (28%) died during ECMO and three (7%) following ECMO.

Thus, the overall ECMO survival rate was 65% (28/43, Figure 1). Over the study time (periods T1–T3) the ECMO survival rates were 65% (17/26), 67% (6/9), 63% (5/8), respectively. During T2 one death and T3 two deaths occurred after de-cannulation. The median duration of ECMO was slightly longer in the survival group (5 vs. 3.5 days), but differences were not significant (*p* = 0.167). Survival in neonates with MAS was 89% (16/18), with CDH 46% (5/11), and septic shock 44% (4/9); thus there was significantly better survival following MAS vs. CDH and septic shock *p* = 0.005 and *p* = 0.006, respectively. Survival rate of miscellaneous diseases was 60% (3/5).

### Ecmo indications over time

ECMO indications changed over time: CDH increased from T1 to T3 from 7.4% to 56% and 50%, respectively (*p* < 0.001), MAS declined from 48 to 33 and 38%, respectively (differences not significant), and EOS declined from 33 to 0 and 0%, respectively (*p* = 0.024). Need for ECMO therapy decreased significantly from T1 to T2 and T3 (*p* < 0.001), respectively, but remained low between T2 and T3.

### ECMO details

Forty-three neonates were treated with ECMO with a median birth weight of 3390 grams (range 1810–4150) and a gestational age of 39 weeks (range 32–43) weeks. Fourteen (32%) neonates were inborn. MAS dominated with 18 cases (41%) followed by CDH (25%), EOS (23%), lung hypoplasia (4.5%), PPHN/PFC (4.5%), and one case of neonatal varicella with respiratory failure (2.3%) (13). Mechanical ventilation started in 42 (95%) on the first day and within the first hour of life in 29 cases (66%). We ventilated with HFO in 64%, initiated iNO in 68%, and gave circulatory support in 64% of the infants. Mean ventilation duration was 45 days. Median OI was 40, FiO2 1.0, and pCO2 86 mmHg. Median ECMO duration was 5 (range 1–30) and length of hospital stay 27 days (3–113). Details on ECMO therapy are given in Table 2, complications on ECMO therapy in Table 3. Shortly, there was a predominance of veno-arterial ECMO (54%) with a median duration of 5 days. Complication rate was 72% (31 of 43 neonates), in 26 cases bleedings, in 15 cases mechanical complications, and in 48 cases different other problems ranging from circulatory deficits (11 cases) to bacterial infections, and from organ failure to cerebral infarction (see Table 3). Any kind of cerebral damage was evident in 9 neonates (20.5%), 5 had cerebral hemorrhages (11.4%) and 4 cerebral infarction (9.1%). In detail cerebral bleeding were mild (IVH grade 1 and 2) in two and severe in three infants with consecutive posthemorrhagic hydrocephalus needing ventriculo-peritoneal shunting. Infarctions were all located in the region of the left mid cerebral artery. One of the latter cases had additional cerebellar hemorrhage.

**Table 2:** Data on ECMO treatment of 43 neonates between 1989 and 2016.

**ECMO parameter**	**Number**
Veno-arterial (VA)	23 (54)
Veno-venous (VV)	17 (39)
VA+VV	3 (7)
Start with ECMO (in hours)	24 (6–1728)
Duration of ECMO (in days)	5 (1–30)
Complications on ECMO	31 (72)
Survivors	28 (65)
*Deaths*	15 (35)
On ECMO	12 (80)
Following ECMO	3 (20)

*Data are given as n (%) or median (range)*.

**Table 3:** Complications (*n* = 89) during ECMO treatment of 31 neonates in Graz, Austria, between 1989 and 2016.

**Complication type**	**Total number**	**Detailed**
*Bleedings*	*26*	
Cannula hemorrhage		6
Lung hemorrhage		6
Surgical hemorrhage		5
Cerebral hemorrhage		5
Gastrointestinal hemorrhage		3
Haematothorax		1
*Mechanical complications*	*15*	
Clotting		5
Oxygenator		4
Pump		3
System stop without cause		2
System change		1
*Other complications*	*48*	
Circulatory deficits		11
Problems with drainages		8
Bacterial infection		8
Capillary-leak-syndrome		5
Cerebral infarction		4
Disseminated intravasal coagulopathy		4
Cardiogenic shock		3
Renal failure		3
Resuscitation on ECMO		1
Unsuccessful start due to prematurity		1

*Data are given as n*.

Deaths included respiratory failure (RF) following ECMO (*n* = 2), multi-organ failure (MOF) following ECMO (*n* = 1), capillary leak and MOF (*n* = 3), septic shock and MOF (*n* = 3), renal failure despite hemofiltration and MOF (*n* = 1), severe lung hypoplasia with RF (*n* = 2), MOF and cardiac arrest (*n* = 1), and brain-death (*n* = 2) with removal of live-sustaining therapy.

### Follow-up

Follow-up rate was 93% (26/28) at a median age of 6 years and 1 month. Seventeen of 26 children (65%) showed normal neurodevelopmental outcome. Motor deficits were present in one case, cognitive deficits in 9 cases (35%). Details are listed in Table 4. Median length of hospital stay was 38.5 days (18–113) in survivors, with 78 days in those with deficits and 29 in those having developed normally (*p* < 0.001). Additionally we observed a trend to lower rates of neurodevelopmental impairment over time (T1 7/16 = 44% vs. T2 1/5 = 20% and T3 1/5 = 20%, *p* = 0.182, respectively). Due to a high rate of lost to follow-up in those children surviving without need for ECMO data are depictured in Table 4 without further analysis.

**Table 4:** Neurodevelopmental follow-up of 47 survivors having been admitted to ECMO treatment in Graz, Austria, between 1989 and 2016.

**Follow-up data**	**ECMO survivors *n* = 28**	**Survivors without ECMO, *n* = 19**
Lost to follow-up	2 (7.1)	10 (53)
Follow-up	26	9
Age (months)	73 (24–276)	9 (3–72)
Normal development	17 (65)	7 (78)
Cognitive/ motor deficits	9 (35)	2 (22)
*Cognitive deficits*	9 (100)	2 (100)
Developmental delay	1 (11)	1 (50)
Mental Retardation	8 (89)	1 (50)
*Motor function deficits*	1 (11)	–
Athetosis	1 (100)	–
Cerebral palsy	0 (0)	–
Microcephaly	3 (11.5)	1 (50)
Dystrophy	1 (3.9)	–
Seizures	1 (3.9)	–
Perception disorder	2 (7.7)	–
Attention deficit syndrome	1 (3.9)	–
Learning difficulties	1 (3.9)	–

*Data are given as n (%) or median (range)*.

## Discussion

Over 28 years we observed a significant decline regarding both referral to and need for ECMO therapy. Interestingly, survival rate (overall 65%) did not change over time. Indications for ECMO changed dramatically over time with EOS declining completely and severe CDH increasing significantly. The use of ECMO for neonatal patients with respiratory failure is worldwide decreasing in proportion to the total annual ECMO runs (as reported by the ELSO registry) most likely due to advancements in medical management and other indications for ECMO (14). We observed a 72% complication rate on ECMO with predominantly hemorrhagic (29%) and mechanical (17%) complications. Outcome revealed two third of the children having developed normally, and the majority of survivors with deficits having cognitive impairment.

Survival rate from the most recent ELSO registry report was 73% in the neonatal population with rates for CDH of 50%, MAS 93%, PPHN/PFC 76%, and for sepsis 72%, respectively (2); in summary comparable to our results. In nearly three quarter the mode of neonatal ECMO was VA (73%), markedly higher than our 54% rate. Trend analysis of survival rates showed either no changes or a decline associated with certain diagnoses including CDH and EOS (15). Survival rates of 54% for infarctions and 44% for hemorrhages have been reported (prevalence rates of 7.2 and 7.1%, respectively) (15). The incidence of abnormalities discovered in neuroimaging during or after ECMO varies from 10% to even 59% according to the literature (16).

In neonates with CDH on ECMO almost one-half (48.1%) survived to discharge, representing a significant decline in survival over the past 25 years (17). This is comparable to our survival rate of 46% in case of CDH over the last 20 years. Thus we agree with the authors (17) that survival rates for neonates with CDH receiving ECMO have continued to drop although the safety of ECMO has improved over the last decade. A recent review found that any benefits of ECMO in CDH remain unclear; and hence leaves the reader with more questions than answers. Answers looked for as regarding prognostication that predicts reversibility of pulmonary hypertension. Criteria for referral for ECMO or optimal timing of surgery for patients on ECMO are still in debate (17). In a prospective observational cohort study of 514 consecutive neonatal and pediatric patients with a survival rate of 55% intracranial hemorrhage was diagnosed in 16% (near to our 11.4% rate) that was independently associated with a higher risk of mortality (18). Chronic conditions, prematurity, VA-ECMO, increased red cell transfusion in the first 24 h of ECMO, and longer ECMO duration were independently associated with mortality in neonates (19).

Intelligence of a population at 8-years follow-up was within normal range with a mean IQ of 99.9, and 91 % of the children followed regular education. Nine percent attended special education, and 39% received extra support in regular education (20). Among neonates, development of renal failure and longer hospitalization were found to be independently associated with worse outcome (20). Longer hospital stay was in our study also a finding associated with developmental delay. A lot of studies report normal cognitive development besides a 4% rate of mental retardation in one and later cognitive problems at school age in some other studies (21). Motor function seems to develop widely normal in most neonatal ECMO survivors over the first years of life with differences being associated with certain diseases, e.g., MAS vs. CDH (21). Risk factors for motor impairment included low socio-economic status, intracranial abnormalities, and long duration of hospitalization. Adult outcomes evaluated by means of a multisite cross sectional survey revealed more physical, mental, and developmental problems (19.9% vs. 10.9%); and more medical complications compared to age-matched national cohorts, but, interestingly, neonatal ECMO survivors were more satisfied with life, more educated and more insured for health care (22). Furthermore, learning problems occurred in near a third of the cohort. The CDH group was generally less healthy and less well educated, but equally satisfied with life. In a nationwide cohort of neonatal ECMO survivors from the Netherlands only children attending special education had below-average intelligence (mean IQ: 76 ± 15); and compared with children with other diagnoses children with CDH scored significantly lower on both IQ and selective attention (23).

A recent single center ECMO experience over 10 years from Taiwan reported 21 neonates with respiratory support and presented comparable results with a survival rate of 62%, and MAS having a superior outcome (11/13, 85%) compared to CDH having the worst one (4/7, 57%) (24). Median ECMO duration and hospital stay for survivors were 6 and 37 days, respectively, and comparable to our findings. We recently reported a significant decline of MAS over 21 years (13), but the proportion of severe cases did not change over time as did not the indication for ECMO. Benefits of ECMO in terms of clinical and cost effectiveness of ECMO compared to conventional ventilatory support for severe respiratory failure in newborn infants have been evaluated by a Cochrane review in 2008 (25) in newborn infants (three US and one UK trials). All trials revealed significant benefit of ECMO on mortality with special regard to neonates without CDH. Data from the UK trial reported additional benefits of ECMO on long-term outcome up to 7 years of age. In addition cost effectiveness of ECMO was comparable to other technologies in common use.

The strengths of the study are the long study period considered for analysis (28 years of ECMO program divided into 3 times spans) and the detailed analysis of indications, complications and outcomes of ECMO and non ECMO patients conducted for each period. Additionally, we provided long-term neurodevelopmental outcome data with high follow-up rates. Limitations of the study include the retrospective data collection and the sample size. Thus, attempts to control for patient population within each era (e.g., race/ethnicity, socioeconomic status) were significantly limited and did not include hospital and provider/team level characteristics, which may also influence referral patterns, candidacy for ECMO, or quality of care (teaching/academic hospital vs. community/private practice model).

In conclusion, we found no significant changes regarding survival rates over the study period besides significant changes in indications for ECMO—with EOS having completely declined and severe CDH having increased while MAS remaining unchanged. Two third of the neonates had normal neurodevelopmental outcome. Cognitive impairment was the major long-term deficit following neonatal ECMO resulting mostly from the first decade of ECMO therapy. Longer hospital stay was associated with worse neurodevelopmental outcome.

## Author contributions

FR and BR were responsible for study planning, design of the study, and writing the manuscript. ER collected all the data and developed the tables. MH did the formal analysis and validated the data. UM-F tested all the children and provided neurodevelopmental follow-up data. GZ analyzed all ECMO data in detail. MR validated the sonographic data and supervised the writing, and interpretation of study findings. BU reviewed and edited the final version of the manuscript.

### Conflict of interest statement

The authors declare that the research was conducted in the absence of any commercial or financial relationships that could be construed as a potential conflict of interest.
